# Ecological Niche Changes and Risk Regionalization of the Invasive Plant *Praxelis clematidea*

**DOI:** 10.1002/ece3.71546

**Published:** 2025-06-18

**Authors:** Jia‐Guo Wang, Jia‐Wei Wu, Wei‐Jie Li

**Affiliations:** ^1^ Guizhou Institute of Mountain Resources Guiyang China; ^2^ Guizhou Key Laboratory of Agricultural Biosecurity Guiyang China; ^3^ Guizhou Botanical Garden Guiyang China

**Keywords:** ecological niche, exotic invasive species, risk pattern, spread trend

## Abstract

*Praxelis clematidea* is a noxious invasive weed. Understanding the dispersion trends and niche changes inherent to *P. clematidea* will be helpful for monitoring this invasive species and for providing early warnings of its spread and developing appropriate scientific prevention and control measures. In this study, the invasion risk zones of *P. clematidea* in Guizhou Province were classified via MaxEnt, Zonation, and ArcGIS. The dispersion trend was predicted, and the ecological niche change was quantified via the R software ecospat package. The results revealed that (1) the current high‐risk areas for *P. clematidea* invasion in Guizhou cover 14,096.03 km^2^, concentrated mainly in the western to southern regions (Liupanshui, Anshun, Qianxinan, and Qiannan); the medium‐risk areas cover 21,144.04 km^2^, concentrated mainly in the southwestern region (Anshun, Qiannan); and the low‐risk areas cover 26430.05 km^2^, occurring in all cities of Guizhou but concentrated mainly in the small areas outside the high‐ and medium‐risk areas. (2) From the present until the 2050s, the risk areas of *P. clematidea* will expand mainly toward the southeastern parts; from the 2050s to the 2070s, the risk areas will decrease in the southeast; and from the 2070s to the 2090s, they will expand at a large scale in the central and northeastern parts. Overall, the trend is toward expansion. (3) The degree of ecological niche overlap between *P. clematidea* in Guizhou Province and its original habitat is very low (Schoener's *D*= 0.12); the rates of niche expansion, stability, and underfilling are 0.88, 0.12, and 0.96, respectively, indicating niche instability. *P. clematidea* invades and occupies areas with relatively high precipitation during the warmest season in Guizhou Province. Compared with the temperature preferences in the coldest season in the original area, this species can adapt to low temperatures.

## Graphical abstract

*Praxelis clematidea* is a noxious invasive weed. Understanding the dispersion trends and niche changes inherent to *P. clematidea* is helpful for monitoring this invasive species and for providing early warnings of its spread and appropriate prevention and scientifically‐based control measures. In this study, the invasion risk zones of *P. clematidea* in Guizhou Province were classified via MaxEnt, Zonation, and ArcGIS.

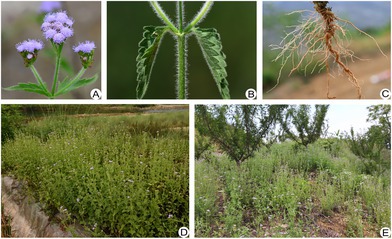

## 1 Introduction

At present, most countries are facing problems with biological invasions (Du et al. 2023). Biological invasions are among the primary driving factors that influence biological species composition, community structure, and ecosystem function (Liu et al. 2022). Biological invasions are also among the main factors leading to the extinction of native species; furthermore, with globalization and future climate change, invasions will become increasingly severe. Invasion risk assessment of exotic species is the most effective and economical measure for preventing biological invasions, and predicting the potential occurrence areas of invasive species is an important part of invasion risk assessment (Li et al. 2022). The ecological niche model (ENM) is a tool used to predict the potential occurrence area of a species and the occurrence probability of a target species in a landscape on the basis of environmental variables and species occurrence sites (Schnase et al. 2021). Several researchers have applied the Zonation tool, which is a spatial optimization tool, to identify the core areas of species invasion and divide potential occurrence areas into risk areas at different levels (Li et al. 2017), further saving management and control costs. Another area of biological invasion research determines whether the niches of invasive species change across time and space. Some scholars believe that the changes in niches during short historical periods are relatively small and that the niches of invasive species are maintained across time and space (Liu et al. 2020). However, other studies have indicated that following the colonization of invasive species in a new environment, great changes in the niche occur, and the niche drifts across time and space (Xian et al. 2023). Although the niche of each invasive species varies with different environments, understanding the changes in the niches of invasive species remains an effective way to determine the adaptability and dispersal limitations of each species.

*Praxelis clematidea* (Figure S1) is an annual herb of the genus *Praxelis* of the family Asteraceae. It is native to South America and was unintentionally introduced through the import of ornamental plants. *Praxelis clematidea* was first discovered in Hong Kong in the 1980s (Jin et al. 2020). This species has large seeds and can be transmitted by wind, water, and humans (Liu 2016). It is salt tolerant (Kan et al. 2009a), drought tolerant (Kan et al. 2009b), and otherwise robust, with strong adaptability and dispersal ability and high genetic diversity (Li et al. 2014; Huang 2014); *P. clematidea* is distributed across more than 10 provinces in China (Qi et al. 2023). This species has strong resource competitiveness. After invading cultivated land, a single dominant community typically forms and consumes a large amount of nutrients, including nitrogen, phosphorus, and potassium, thereby reducing soil fertility; additionally, these plants often surround other plants, and their root exudates can be toxic to surrounding plants. This occurrence results in reduced crop yield and reduced biodiversity (Li et al. 2006). *Praxelis clematidea* is considered a noxious invasive weed because it spreads quickly and severely damages the agricultural economy and biodiversity (Yan et al. 2014); it has been included on the “List of Invasive Species under Key Management” in China. *Praxelis clematidea* was introduced to China more than 40 years ago, and many studies have focused on this topic in China; however, studies on the spatiotemporal variations in the ecological niches of *P. clematidea* and invasion risk areas have not been reported. During our field survey, the authors found that *P. clematidea* had invaded habitats, such as orchards, tea gardens, dry land, rural roads, highway land, and village land, in more than 20 counties of Guizhou Province, resulting in a certain degree of invasion. However, little research has been conducted on *P. clematidea* in the province, and relevant studies are rare. Therefore, this study took Guizhou Province as the study area, and on the basis of the information of the *P. clematidea* distribution sites in Guizhou Province and their origin, the quantification of niches and the prediction of risk areas were performed. The purposes of this study were to investigate the following aspects: (1) After the invasion of Guizhou Province, how would the niches change? (2) Which areas are the key areas of invasion by *P. clematidea*? (3) How did the spatial pattern of the key invasion areas change over time? The spatiotemporal changes in the niches and invasion risk areas of *P. clematidea* have been clarified to provide references for monitoring, early warning, control and management.

## 2 Materials and Methods

### 2.1 Overview of the Study Area

Guizhou Province is located in the karst area of southwestern China on the Yunnan–Guizhou Plateau, with geographic coordinates of 103°36′–109°35′ E and 24°37′–29°13′ N; has a land area of approximately 176,200 km^2^; and is composed predominantly of three fundamental types of landforms: plateau mountains, hills, and basins, with mountainous and hilly terrain collectively occupying 92.5% of the total area. The climate is generally a subtropical humid monsoon climate and mild and humid, with an average annual temperature of 14°C–16°C in most areas. The precipitation is concentrated mainly between April and September, and the annual precipitation is generally 1100–1400 mm, showing a spatially decreasing trend from southeast to northwest (Pan et al. 2023; Wang et al. 2022).

### 2.2 Distribution Data

The distribution site data of *P. clematidea* in Guizhou Province were collected by our team through field surveys conducted from April 2022 to December 2023. Data collection was conducted at the county‐level, with sampling routes set as evenly as possible to the east, south, west, north, and center of each county. Sampling points were established along each route on the basis of the presence or absence of *P. clematidea*. The sampling routes covered all 1509 township‐level administrative regions under the 88 county‐level administrative regions with 9 prefecture‐level cities in Guizhou Province, focusing primarily on habitats with high invasion rates, such as dry lands, orchards, barren slopes, and roadside areas.

**FIGURE 1:**
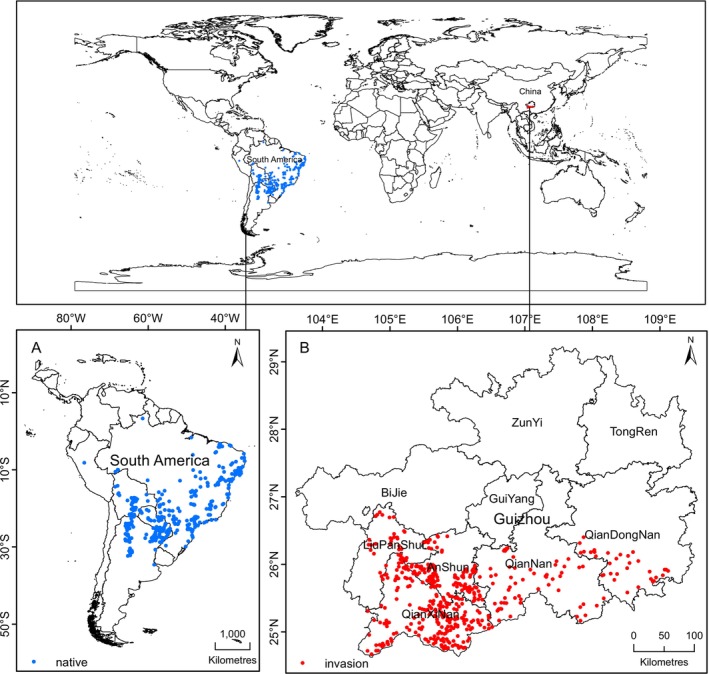
Location information of *Praxelis clematidea*. (A) South America (native). (B) Guizhou Province, China (invaded).

Distribution site data of origin (South America) were obtained from the Global Biodiversity Information Facility (GBIF, https://www.gbif.org/). To address issues related to spatial sampling bias, spatial thinning of species occurrence records was performed via the Thin function from the R package spThin, selecting an interval distance of 1 km (Aiello‐Lammens et al. 2015). This process resulted in 454 invasion points in Guizhou Province and 444 native occurrence points (Figure 1).

### 2.3 Environmental Variables

In South America, *P. clematidea* grows at elevations ranging from 0 to 3050 m. In Hong Kong and Queensland, however, it is found at elevations of 0–700 and 0–800 m, respectively. Furthermore, in Australia and Hong Kong, *P. clematidea* shows a preference for areas with high rainfall and exhibits some tolerance to both high and low temperatures. However, in South America, no frost resistance has been reported for *P. clematidea* (Veldkamp 1999).

On the basis of previous research (Chen, Xian, et al. 2023; Chen, Zhang, et al. 2023; Su et al. 2022), we selected environmental variables that are likely to influence its distribution: (1) historical bioclimatic variables (average values from 1970 to 2000) and future bioclimatic variables (average values for the periods 2021–2040, representing the 2030s; 2041–2060, representing the 2050s; 2061–2080, representing the 2070s; and 2081–2100, representing the 2090s). These variables were derived from the BCC‐CSM2‐MR model under the SSP126 socioeconomic path, which has been demonstrated to reliably simulate precipitation and temperature patterns in China (Wu et al. 2019). The data were sourced from the WorldClim database (http://www.worldclim.org/); (2) topographic variables, including aspect, slope, and elevation, with data obtained from the Digital Geospatial Data Cloud of the Chinese Academy of Sciences (http://www.gscloud.cn); and (3) human activity variables, particularly the human footprint index, with values ranging from 0 to 50, where higher values indicate greater intensity of human activity. These data were sourced from SEDAC (http://sedac.ciesin.columbia.edu/wildareas/). All three groups of environmental variables were resampled in ArcGIS to achieve a uniform raster size of 30 arc‐seconds and a consistent coordinate system of WGS‐1984.

Collinearity among environmental variables can reduce the accuracy of model predictions (De Marco Jr and Nóbrega 2018). To address this issue, Pearson correlation analysis was conducted on the aforementioned three groups of environmental variables (Figure S2). For each set of highly correlated variables (|*p*| > 0.8), only the variable with the highest contribution was retained (Gao et al. 2023). The resulting environmental variables, as shown in Table 1, were converted to the ASCII format in ArcGIS for subsequent use in MaxEnt analysis.

**TABLE 1:** Environmental variables after screening.

Types	Index code	Variable description
Bioclimatic variables	Bio2	Mean diurnal range (°C)
	Bio3	Isothermality (×100)
	Bio11	Mean temperature of coldest quarter (°C)
	Bio12	Annual precipitation (mm)
	Bio14	Precipitation of driest month (mm)
	Bio18	Precipitation of warmest quarter (mm)
Topographic variables	Asp	Aspect
	Slo	Slope
	Ele	Elevation (m)
Human activity variables	Hfi	Human footprint index

### 2.4 MaxEnt Modeling

MaxEnt is one of the most popular tools for species distribution and ecological niche modeling, primarily due to its high predictive accuracy (Merow et al. 2013). It employs a machine learning method based on maximum entropy modeling to construct ecological niche models (ENMs), allowing researchers to estimate the potential distribution of a species using occurrence data and a set of environmental variables (Schnase et al. 2021). This study employs the MaxEnt model to predict the potential geographic distribution of *P. clematidea* in Guizhou Province, China. The software was obtained from https://biodiversityinformatics.amnh.org/open_source/MaxEnt/. Typically, ecological niche modeling with MaxEnt utilizes the software's default settings. However, these defaults may not be optimal for certain species (Merow et al. 2013).

This study leveraged the R package ENMeval (Muscarella et al. 2014) to adjust the regularization multiplier (RM, a form of L_1_‐regularization) and feature combination (FC) (Zhang, Chen, et al. 2023). FC controls the shape of the covariate‐species relationships and RM penalizes each covariate by L_1_‐regularization. By analyzing model complexity across different parameter combinations, we selected the combination with lower complexity for modeling and thus optimized the model. As our model compares the environmental conditions of occurrence locations with those of background locations, it is necessary to randomly sample points from the background area. We defined the background by generating 10,000 points within Köppen‐Geiger climate zones containing at least one GPS record (Webber et al. 2011). In addition, traditional random cross‐validation techniques may underestimate the prediction error and lead to inappropriate model selection (Valavi et al. 2019). Therefore, we performed k‐fold cross‐validation by partitioning the occurrence locations into training and test folds using the built‐in functionality of ENMeval. In this study, the RM parameter was set between 1 and 4, with intervals of 0.5, resulting in a total of 8 RM parameters. For the FC parameter, the MaxEnt model provides five features (Elith et al. 2011): linear (L), quadratic (Q), hinge (H), product (P), and threshold (T). We selected six feature combinations: H, L, LQ, LQH, LQHP, and LQHPT. Using the ENMeval package (Muscarella et al. 2014), we employed the following criteria. (1) The Akaike information criterion correction (AICc) was used to select the best model, with the optimal model having the lowest AICc value (i.e., ΔAICc = 0). (2) The difference between the training and test areas under the receiver operating characteristic curve (AUC.DIFF) and the 10% training omission rate (OR10) was used to evaluate the degree of model overfitting. OR10 was used to evaluate the model's prediction error with regard to known occurrence points. Specifically, OR10 refers to the allowance that 10% of known occurrence points in the training data may be predicted as “unsuitable” by the model. For an ideal model, this value should be approximately 0.1 (Hwang et al. 2022). AUC.DIFF (AUC_train—AUC_test) was used to measure the difference in the model's performance between the training and testing datasets. Large AUC.DIFF and high OR10 values typically indicate model overfitting, meaning that the model performs well on the training data but fails to generalize effectively to independent test data. (3) The area under the receiver operating characteristic curve (AUC) was used to measure model accuracy, with AUC ∈ [0.80–0.90) indicating good predictive performance and AUC ∈ [0.90–1.0) indicating excellent predictive performance (Fang et al. 2024). Finally, we selected the parameter combination corresponding to ΔAICc = 0 to establish the optimized model.

### 2.5 Division of *P. clematidea* Risk Areas

Zonation is widely used for landscape prioritization, as it aims to identify ecologically significant (or insignificant) areas (Moilanen et al. 2011). The outputs of the Zonation software include priority maps and performance curves, with the latter depicting the relationship between the proportion of landscape loss and the proportion of species remaining. Landscapes that retain a relatively high proportion of species are generally considered to have relatively high priority (Lehtomäki and Moilanen 2013). In this study, we employed Zonation v4.0 software to prioritize the invasion landscape of *P. clematidea*, categorizing landscapes with different invasion priorities into various levels of invasion risk areas. Specifically, we divided the invasion landscape of *P. clematidea* into high‐risk, medium‐risk, low‐risk, and non‐risk areas according to their priority order, with the goal of facilitating more effective management of the species.

Zonation prioritizes landscapes via different algorithms. We used the potential geographic distribution generated by MaxEnt (in ASC format) as the input and selected the Core Area Zonation (CAZ) algorithm for spatial prioritization, setting the warp factor to 1. We then ran Zonation software (Lehtomäki and Moilanen 2013).

### 2.6 Prediction of the Changes in the *P. clematidea* Risk Area

Risk areas are typically regions where invasive species initially establish populations and are most susceptible to outbreaks and spread. Predicting future trends in the risk areas of *P. clematidea* is essential for effective monitoring and early warning systems.

In this study, we forecasted the future trends of these risk areas. The prediction process involved the following steps: (1) Using environmental variables from different periods, we predicted the potential geographic distributions of *P. clematidea* for each period and delineated the risk areas on the basis of the invasion landscape prioritization for each period; (2) we then utilized the SDMtoolbox (http://www.sdmtoolbox.org/) to compare the risk areas across different periods and identify trends in the changes in these risk areas.

### 2.7 Quantification of the Niche of *P. clematidea*

On the basis of the distribution points of *P. clematidea* in its native range and in Guizhou Province, as well as environmental variables, the ecospat package was utilized to evaluate the niche stability (NS), niche expansion (NE), and niche underfilling (NU) of *P. clematidea* following its invasion into Guizhou (Di Cola et al. 2017). NS denotes the ecological niche occupied in both the native and invaded ranges; NE indicates the ecological niche newly occupied in the invaded range; and NU represents the niche that is unoccupied in the invasive range relative to the native range (Wei et al. 2022; Guisan et al. 2014). Additionally, we conducted a PCA by combining the background values for all the environmental variables from both the native and invasive ranges of *P. clematidea*, which is essential for niche quantification. Furthermore, this study included calculations of Schoener's *D*, niche equivalency tests, and niche similarity tests. Schoener's *D* measures the degree of overlap between the ecological niches of the native and invaded ranges, with values ranging from 0 to 1, where 0 signifies no overlap and 1 signifies complete overlap. The niche equivalency test examines whether the ecological niches in the native and invaded ranges are equivalent, whereas the niche similarity test assesses whether the overlap of the native niche with the invaded niche is greater than would be expected by random distribution (Gama et al. 2017; Warren et al. 2008).

## 3 Results

### 3.1 Evaluation of the Prediction Accuracy of the MaxEnt Model

**FIGURE 2:**
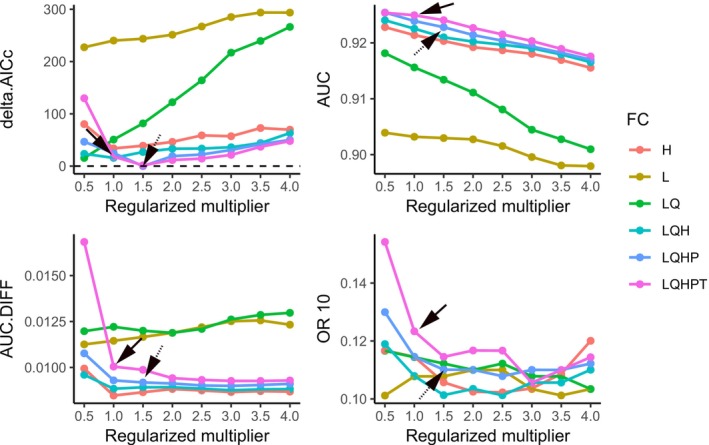
MaxEnt evaluation metrics generated by ENMeval. The solid arrows indicate the default parameters of the MaxEnt model, whereas the dashed arrows indicate the optimized model parameters.

As shown in Figure 2, the optimization results indicate that setting the RM to 1.5 and the FC to LQHP yields a ΔAICc of 0, identifying this combination as the optimal configuration for the MaxEnt model in this study. Both the AUC.DIFF and OR10 metrics of the optimized model are lower than those of the model using default parameters, suggesting that the optimized model reduces overfitting to the distribution data. Additionally, the AUC value of the optimized model is 0.9228, reflecting excellent predictive performance.

### 3.2 Evaluation of the Importance of Environmental Variables for the Potential Distribution of *P. clematidea* in Guizhou

According to the MaxEnt model evaluation results (Table 2), two primary environmental variables influence the current potential geographic distribution of *P. clematidea* in Guizhou: the precipitation of the warmest quarter (Bio18) and the mean temperature of the coldest quarter (Bio11), with contribution rates of 54.1% and 23.7%, respectively. The combined contribution rate of these two environmental variables reached 77.8%, indicating that the distribution of *P. clematidea* is affected mainly by temperature and precipitation. Additionally, the contribution rates of the nonprimary environmental variables are all less than 7%, which is significantly lower than those of the above two primary environmental variables. The two most important environmental variables in terms of permutation importance are precipitation in the warmest quarter (Bio18) and elevation (Ele), with values of 43.7% and 21.5%, respectively.

**TABLE 2:** Contribution rates of different environmental variables to the current potential geographic distribution of *Praxelis clematidea* in Guizhou.

Types	Index code	Percent contribution (%)	Permutation importance (%)
Bioclimatic variables	Bio2	1	5.9
	Bio3	6.9	7.8
	Bio11	23.7	10.6
	Bio12	2.4	4.5
	Bio14	0.6	1.8
	Bio18	54.1	43.7
Topographic variables	Asp	0.8	1.0
	Slo	1.7	2.1
	Ele	6.8	21.5
Human activity variables	Hfi	1.9	1.2

### 3.3 Invasion Risk Zoning

**FIGURE 3:**
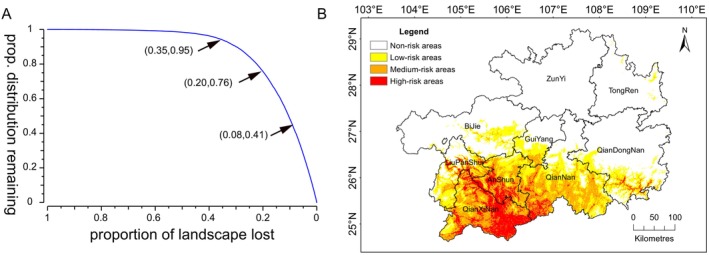
(A) The zonation performance curve. (B) The current invasion risk zones of *Praxelis clematidea*.

The Zonation performance curves (Figure 3A) indicate that when the total landscape loss ranges from 8% to 0%, the remaining proportion of *P. clematidea* decreases from 0.41 to 0. This suggests that this continuous landscape has the highest priority and is considered a high‐risk area for *P. clematidea* invasion. When the total landscape loss ranges from 20% to 8%, the remaining proportion of *P. clematidea* decreases by 0.35%, indicating that this continuous landscape has the second highest priority and is considered a medium‐risk area. When the total landscape loss ranges from 35% to 20%, the remaining proportion of *P. clematidea* decreases by 0.19%, suggesting that this continuous landscape has the third highest priority and is considered a low‐risk area. When the total landscape loss ranges from 100% to 35%, the remaining proportion of *P. clematidea* shows almost no change, indicating that this continuous landscape has the lowest priority and is considered a non‐risk area for *P. clematidea* invasion.

As shown in Figure 3B and Table 3, the high‐risk areas include regions, such as Qianxinan, Anshun, and Qiannan and parts of Liupanshui and Qiandongnan adjacent to these regions, with a total area of 14,096.03 km^2^. These areas are primarily concentrated in southwestern Guizhou, which is the current typical occurrence area of *P. clematidea*. The medium‐risk areas are located at the periphery of the high‐risk areas and include regions from Liupanshui to Anshun, Qiannan, and Qiandongnan, which are concentrated primarily in the Anshun and Qiannan areas; these areas are considered the forefront of the spread, with a total area of 21,144.04 km^2^. The low‐risk areas cover a broader range than do the high‐ and medium‐risk areas. These low‐risk areas include parts of Bijie (central to southern), Tongren (eastern), and Qiandongnan (southern), which are considered potential risk areas with a total area of 26,430.05 km^2^. The total area of high‐, medium‐, and low‐risk areas is 61,670.12 km^2^, accounting for 35% of Guizhou's total land area. The remaining areas are considered non‐risk areas, which account for a total area of 114,530.22 km^2^.

**TABLE 3:** Areas of different risk zones.

Risk area level	Area (km^2^)	Proportion of Guizhou's land area (%)
High‐risk areas	14,096.03	8
Medium‐risk areas	21,144.04	12
Low‐risk areas	26,430.05	15
Non‐grade risk areas	114,530.22	65

### 3.4 Forecast of Changes in the Risk Area

**FIGURE 4:**
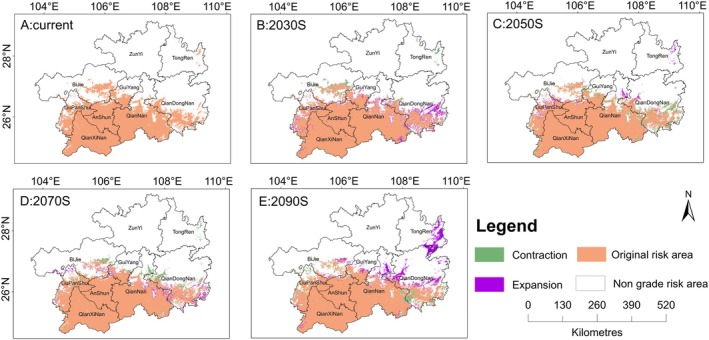
Prediction of changes in the risk area of *Praxelis clematidea*. (A) The current risk area for the invasion of *P. clematidea*; (B–E) represent the trend of changes in the risk area of *P. clematidea* from the 2030s to the 2090s.

As shown in Table 4 and Figure 4, compared with the current situation, the risk zone area for *P. clematidea* in the 2030s is projected to increase by 2041.15 km^2^, expanding primarily in southeastern Qiandongnan. From the 2030s to the 2050s, the risk zone area is expected to decrease by 1727.49 km^2^, contracting mainly in southeastern Qiandongnan. Between the 2050s and 2070s, the risk zone area is projected to decrease by 499.30 km^2^, contracting primarily in Qiannan and western Qiandongnan. From the 2070s to the 2090s, the risk zone area is expected to increase by 4017.30 km^2^, with expansion primarily concentrated in Tongren and Qiandongnan. By the 2090s, the risk zone for *P. clematidea* could occupy over half of Guizhou Province. Overall, the future trend shows an increase in the risk zone, with a total expansion area projected to be 3831.66 km^2^.

**TABLE 4:** Future changes in the areas at risk for *Praxelis clematidea*.

Year	Risk zone area (km^2^)	Increase/decrease (km^2^)	Trend of change
Current	61,670.12	—	—
2030s	63,711.27	2041.15	Expansion
2050s	61,983.78	−1727.49	Contraction
2070s	61,484.48	−499.30	Contraction
2090s	65,501.78	4017.30	Expansion
Total	—	3831.66	Expansion

### 3.5 Niche Changes

**FIGURE 5:**
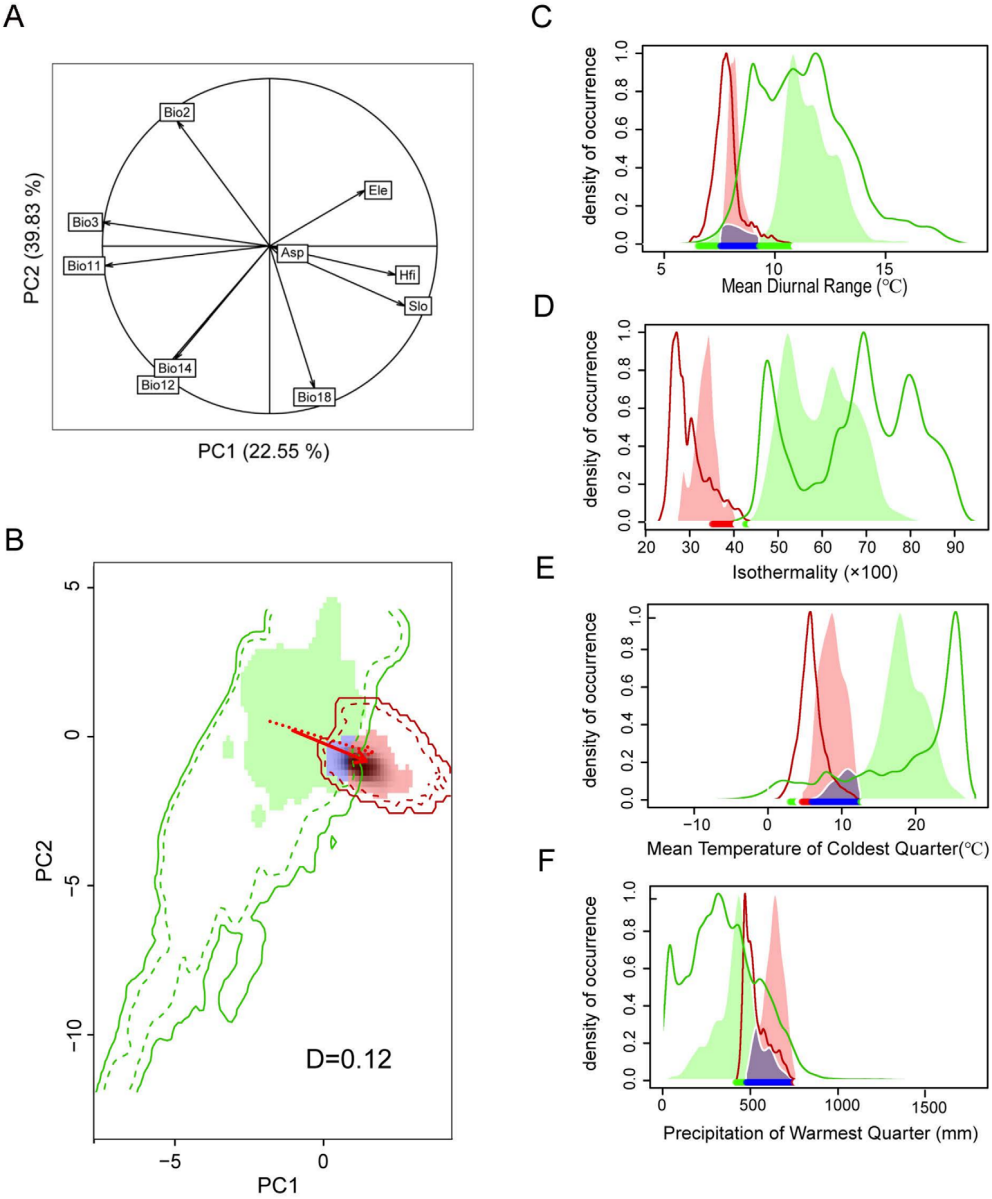
Niche results of *Praxelis clematidea* based on principal component analysis (PCA). (A) Principal component analysis of all background environmental variables. (B) Niche overlap between the native and invaded ranges, with a Schoener's *D* value of 0.12; the green area represents the niche occupied by *P. clematidea* in its native range, the pink area represents the niche in the invaded range, and the blue area indicates the overlapping niche; solid and dashed lines represent 100% and 50% of the available background environment, respectively; solid arrows and dashed arrows indicate the shifts in the niche and background environment centroids, respectively. (C–F) Univariate niche distribution characteristics, with colors and solid lines representing the same meanings as in (B); the shaded area represents the overlapping niche between the native range and Guizhou.

Principal component analysis (PCA) of the background values for all the environmental variables (Figure 5A) revealed that PC1 and PC2 explain 39.83% and 22.55% of the total variability, respectively, totaling 62.38%. PC1 is associated mainly with Bio3 (isothermality) and Bio11 (mean temperature of coldest quarter), whereas PC2 is correlated with Bio2 (mean diurnal range) and Bio18 (precipitation of warmest quarter). These results, combined with the results of the ecological niche overlap analysis (Figure 5B), indicate that *P. clematidea* has expanded its ecological niche along the PC1 axis in Guizhou, with very little overlap with its native range (*D* = 0.12). Single‐factor ecological niche distribution characteristics indicate that *P. clematidea* in Guizhou tends to invade areas with lower monthly temperature ranges and isothermality, both of which show unimodal changes (Figure 5C,D). Although the average temperature of the coldest quarter niche exhibited three density peaks, the high‐density peaks were all at lower temperatures (Figure 5E). *Praxelis clematidea* in Guizhou tends to occupy areas with higher precipitation in the warmest quarter, with density showing unimodal changes as precipitation increases (Figure 5F). The single‐factor ecological niche occupied by *P. clematidea* in Guizhou differs significantly from its native range, with a low degree of overlap.

Similarity and equivalency tests (Table 5) indicate that *P. clematidea* occupies similar but not identical ecological niches between the two regions. Compared with those in the original area, after the invasion of Guizhou Province, 0.88 of the niches of *P. clematidea* expanded, 0.96 of the niches were missing, and only 0.12 of the niches remained stable, indicating rapid and nonconservative expansion of its ecological niche in Guizhou.

**TABLE 5:** Comparison of ecological niches between the production areas of *Praxelis clematidea* and Guizhou Province.

Niche expansion	Niche stability	Niche underfilling	Niche equivalency	Niche similarity
0.88	0.12	0.96	0.01	0.02

## 4 Discussion

*Praxelis clematidea* in Guizhou exhibits an unstable ecological niche and significant expansion, which may be attributed to adaptive evolution in response to different environments. This adaptive strategy contributes to its strong adaptability and high invasiveness. Considering its dispersal characteristics, future changes in the ecological niche and invasion risk pattern of *P. clematidea* in Guizhou, as well as effective prevention and control measures, are important.

### 4.1 The Niche of *P. clematidea* Is Not Conserved; It Is Expanding Significantly

Some scholars believe that geographic, environmental, and climatic conditions similar to those of an area of origin are often more conducive to establishing populations of invasive species; therefore, the niches of invasive species are more likely to be conserved. The niche conservation hypothesis is widely used to explain changes in the niches of invasive species (Liu et al. 2020). However, the niche conservation hypothesis is not suitable for all invasive species. Owing to biotic interactions (including predation and competition), changes in geographic restriction, or adaptive evolution, some invasive species can occupy other, different ecological environments, causing niche drift to occur (Song et al. 2023). For example, *Ageratina adenophora* , also known as Crofton weed, is a plant species in the family Asteraceae. After *A. adenophora* invaded China from Mexico, its ecological niche expanded (Xian et al. 2023); similarly, after invading Asia, Africa, and South America from Mexico, *Leucaena leucocephala* exhibited significant differences in its ecological niche compared with its native region (Ahmad et al. 2019). Niche shifts might be adaptations to local environments, increasing their invasive potential within the region (Xian et al. 2023).

*Praxelis clematidea* are highly invasive plants that have invaded many provinces in China. Previous studies have conducted ISSR analysis on eight *P. clematidea* populations from Hainan and Guangdong Provinces. The results showed that *P. clematidea* had high genetic diversity, indicating that *P. clematidea* underwent adaptive evolution in response to new environments (Li et al. 2014); a study on the genetic diversity of *P. clematidea* populations in 15 cities in Hainan, Guangdong, and Fujian Provinces also confirmed this interpretation (Huang 2014). Niche equivalency and niche overlap analyses revealed that *P. clematidea* are not equivalent in the native range (South America) and invaded range (Guizhou), with a 0.96 niche loss relative to its native range and a 0.88 niche expansion. These results indicate that *P. clematidea* has undergone niche differentiation. The univariate niche distribution characteristics revealed that in both its native area and invasion area (Guizhou Province), *P. clematidea* preferred to disperse toward high‐temperature areas. However, notably, the preference for high temperatures in Guizhou Province was lower than that in the original area, which indicates that *P. clematidea* may be gradually adapting to low temperatures in China. Although the ecological niche of *P. clematidea* has undergone differentiation compared to its native range after its invasion of Guizhou, *P. clematidea* has been invading Guizhou for decades; however, we have not yet observed its presence in the central or northern regions of Guizhou. Whether its ecological niche in Guizhou has reached equilibrium remains uncertain.

### 4.2 Formation and Spatiotemporal Changes in the *P. clematidea* Risk Pattern

The MaxEnt model is a tool for constructing niche models. This approach is advantageous because of its high accuracy and is now widely used for predicting the potential occurrence areas and dispersal trends of organisms (Gao et al. 2023; Zhang, Chen, et al. 2023). The construction of the niche model was based on the distribution sites of the species. One of the key factors in the accuracy of model prediction is whether the obtained distribution sites of species can reflect the distribution range of the species (Zhu et al. 2013). In this study, the sampling coverage of *P. clematidea* distribution points in Guizhou Province was extensive, and these points effectively reflected its current distribution. The MaxEnt model performs well in predicting the distribution areas of *P. clematidea*.

Biological invasion is an orderly ecological process of “introduction‐colonization‐latency‐spread and outbreak” (Chen 2022); when conditions are correct, it may create a disastrous situation (Xu et al. 2004). Clarifying the invasion risk areas of species plays an important role in monitoring these species and supporting early warning systems. The formation of an invasion risk area is related to the ecology, biological characteristics, environment, and invasion history of the invasive species (Chen, Xian, et al. 2023; Chen, Zhang, et al. 2023; Wang et al. 2017; Zhang, Miao, et al. 2023). The seeds of *P. clematidea* exhibit extremely strong transmission ability (Wu et al. 2008). Following the first discovery of *P. clematidea*, this species was found to be widespread in Hainan, Fujian, Guangdong, Guangxi and other provinces (Wang et al. 2006; Zhu et al. 2005), as on the southwestern part of the Guizhou border in Guangxi and Yunnan, *P. clematidea* can spread naturally from Guangxi to southwestern Guizhou. The hot and humid environment in southwestern Guizhou provides good conditions for the growth and development of *P. clematidea*, which colonized the southwestern part of Guizhou Province and gradually spread to the southern and southeastern regions, thus leading to a pattern of invasion risk that gradually decreased from south to north and from west to east. Global warming has a certain impact on the distribution of species, with an increase in suitable areas for most invasive species (Yang et al. 2023). From the present to the 2090s, the invasion risk area of *P. clematidea* in Guizhou Province is expected to gradually increase, with an expanding trend toward central and northeastern Guizhou. Although there is a projected decrease in the invasion risk area by the 2050s and 2070s, overall, the invasion risk area of *P. clematidea* is increasing.

### 4.3 Prevention and Control Strategies

There are few reports on *P. clematidea* in Guizhou Province; however, this plant has spread on a large scale across southwestern Guizhou Province. The area at risk of *P. clematidea* invasion will further increase in the future, and it is difficult to comprehensively curb its proliferation in the short term. Therefore, the author suggests that prevention and control measures for *P. clematidea* should prioritize the front line of spread and potential risk areas, specifically in medium‐ and low‐risk areas. Second, ongoing efforts should focus on controlling outbreaks in typical occurrence areas, aiming to limit threats within the current scope of high‐risk areas and prevent further spread. For invasive high‐risk areas, prevention and control measures should focus on the following: (1) *P. clematidea* should be manually or mechanically removed before seed maturity (before May). (2) Chemical control for *P. clematidea* should include spraying with the single agent glufosinate‐ammonium or glyphosate‐isopropylamine salt + 2 M‐4‐sodium complex (Jin et al. 2020). (3) Biological control should include *Ascomycotina* sp., which can be used to induce disease with *P. clematidea* (Wang et al. 2007), and *Praxelis* witches' broom phytoplasma (PrWB), which can inhibit growth and reproduction (Yang et al. 2012); furthermore, native plants can be selected to replace and resist *P. clematidea*, enhancing shading to improve suppression rates by planting high‐density woody plants that are taller than *P. clematidea*. In areas of moderate risk, emphasis should be placed on conducting regular monitoring 1–2 times per year, providing timely early warnings and forecasts, and implementing real‐time prevention and control measures to interrupt transmission. Furthermore, based on the actual expansion range obtained from multiyear monitoring, it is possible to verify whether the ecological niche of this species in Guizhou is undergoing continuous expansion or approaching equilibrium. In low‐risk areas, emphasis should be placed on patrols. Exploiting the mountainous terrain and geographical features of Guizhou, natural and artificial mixed interception zones can be constructed in the frontier and transitional areas where *P. clematidea* may spread, particularly in key areas, such as traffic arteries, county‐level river entrances, large farmland areas, and regular manuals, barren hills, and slopes. Regular manual inspections should be conducted onsite to promptly eradicate any occurrences, preventing further spread.

## Author Contributions

**Jia‐Guo Wang:** conceptualization (lead), project administration (equal), writing – original draft (lead), writing – review and editing (equal). **Jia‐Wei Wu:** writing – original draft (supporting), writing – review and editing (equal). **Wei‐Jie Li:** conceptualization (supporting), writing – review and editing (equal).

## Conflicts of Interest

The authors declare no conflicts of interest.

## Supporting information


Figure S1.
**Figure S2**.

Data S1.

## Data Availability

All of the distribution site data of *Praxelis clematidea* used in this study are available and are provided in the Supporting Information.
