# Supplementary material for: Ecological Niche Changes and Risk Regionalization of the Invasive Plant Praxelis clematidea
Source: Ecol Evol. 2025 Jun 18;15(6):e71546. doi: 10.1002/ece3.71546 (PMC12176453; doi:10.1002/ece3.71546)
Supplement: Supplementary file 1 — Figure S1. Figure S2. [file ECE3-15-e71546-s001.docx]

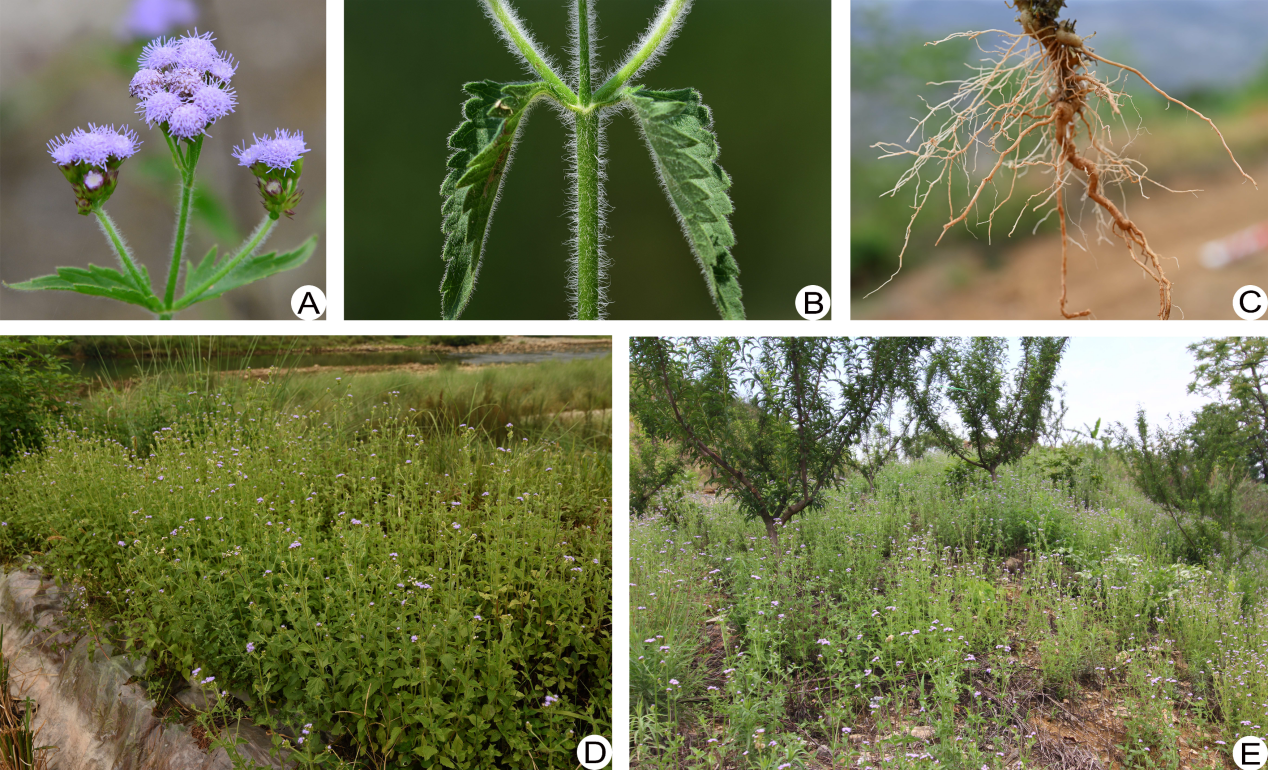


Figure S1 plant morphology and hazards of *P. clematidea*. A: Flowers. B: Stem. C: Root. D: Population. E: Endangering local economic fruit trees.


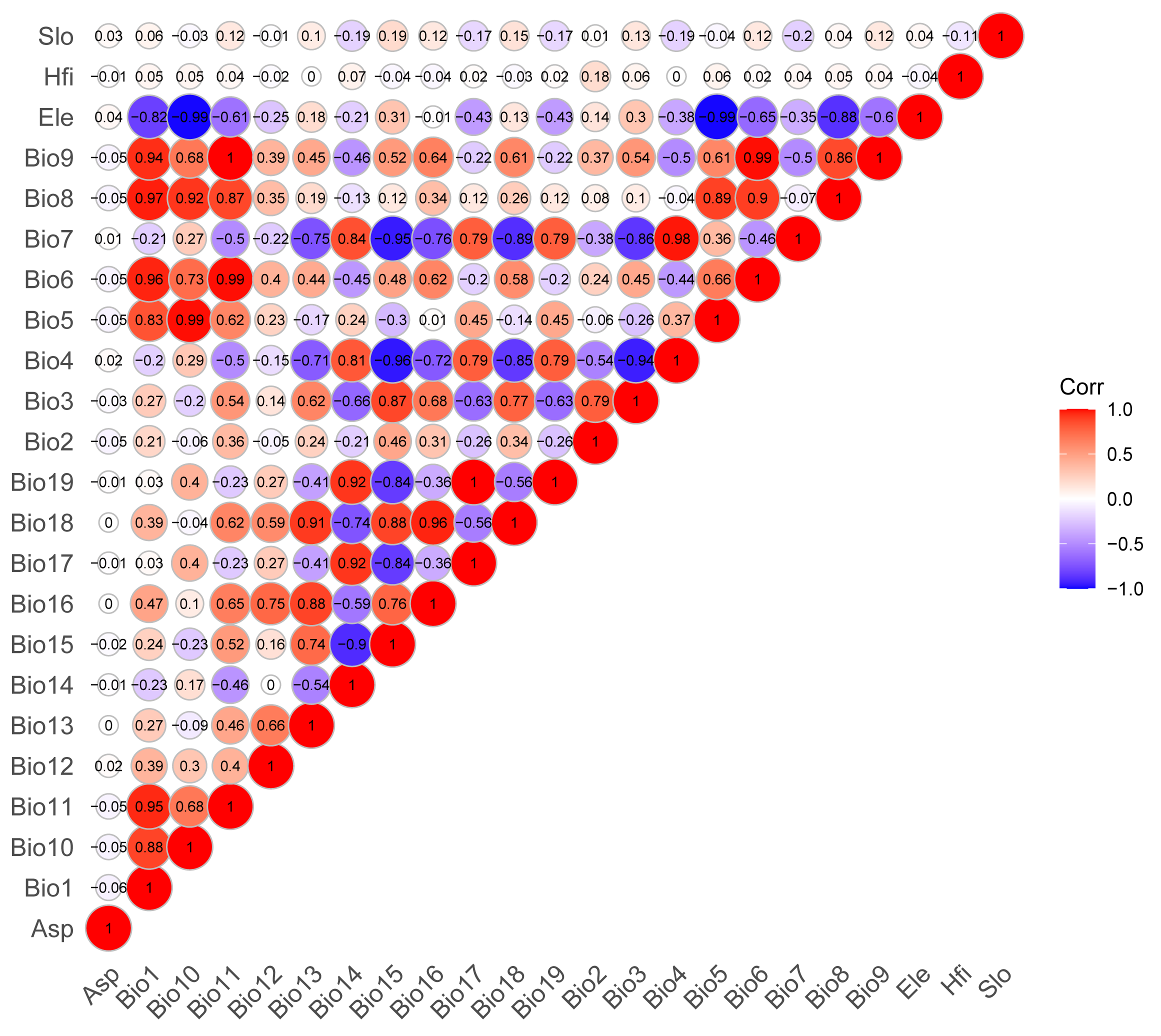


FIGURE S2 Correlation heatmap of the environmental variables
